# A novel application of Lobatto IIIA solver for numerical treatment of mixed convection nanofluidic model

**DOI:** 10.1038/s41598-021-83990-8

**Published:** 2021-02-24

**Authors:** Iftikhar Ahmad, Tahir Nawaz Cheema, Muhammad Asif Zahoor Raja, Saeed Ehsan Awan, Norma Binti Alias, Sana Iqbal, Muhammad Shoaib

**Affiliations:** 1 Department of Mathematics, University of Gujrat, Gujrat, Pakistan; 2 Future Technology Research Center, National Yunlin University of Science and Technology, 123 University Road, Section 3, Douliou, Yunlin 64002 Taiwan, R.O.C.; 3 Department of Electrical and Computer Engineering, COMSATS University Islamabad, Attock Campus, Attock, Pakistan; 4 Center for Sustainable Nanomaterials, Ibnu Sina Institute for Scientific and Industrial Research, Universiti Teknologi Malaysia, UTM Skudai, 81310 Johor, Malaysia; 5 Department of Mathematics, COMSATS University Islamabad, Attock Campus, Attock, Pakistan

**Keywords:** Physics, Fluid dynamics

## Abstract

The objective of the current investigation is to examine the influence of variable viscosity and transverse magnetic field on mixed convection fluid model through stretching sheet based on copper and silver nanoparticles by exploiting the strength of numerical computing via Lobatto IIIA solver. The nonlinear partial differential equations are changed into ordinary differential equations by means of similarity transformations procedure. A renewed finite difference based Lobatto IIIA method is incorporated to solve the fluidic system numerically. Vogel's model is considered to observe the influence of variable viscosity and applied oblique magnetic field with mixed convection along with temperature dependent viscosity. Graphical and numerical illustrations are presented to visualize the behavior of different sundry parameters of interest on velocity and temperature. Outcomes reflect that volumetric fraction of nanoparticles causes to increase the thermal conductivity of the fluid and the temperature enhances due to blade type copper nanoparticles. The convergence analysis on the accuracy to solve the problem is investigated viably though the residual errors with different tolerances to prove the worth of the solver. The temperature of the fluid accelerates due the blade type nanoparticles of copper and skin friction coefficient is reduced due to enhancement of Grashof Number.

## Introduction

The suspension of nanoparticles into the base fluid to increase the thermal conductivity and rate of heat transfer is termed as nanofluid. The term mixed convection is used for the transfer of heat. In the last few decades, it is observed that nanofluids are vastly used to increase the thermal conductivity of the traditional fluids i.e. water, machine oil, fuel oil etc. The uses of nanofluids are in nanotechnology, automatic cooling, temperature exchanger and targeted medication transfer etc. Innovative work by Choi and Eastman conclude that the thermal conductivity of the base fluid can be improved by adding the small number of nanoparticles^1^. Metals, oxides, nitrates and carbides are mostly used nanoparticles. Only 1–5% amount of nanoparticles is added to the base fluid to improve the thermal conductivity of the base fluid. After these preliminary considerations, many investigations have been performed for the nanofluidic problems along with transfer of heat. Advanced heat conduction is important for nanofluids due to large surface area of nanoparticles which permits for additional heat transfer^2^.

The important flow phenomenon arises in the field of boundary layer flow problems along with its extending surface. Aerodynamics, plastic sheet extrusion and artificial extrusion are some of the applications for different fluids. Initially, Sakiadis^3^ analyzed the boundary layer singularity on a solid plate. Crane^4^ observed the flow over a parallel stretching sheet. Later, many researchers worked on the flow for extended as well as moving surfaces under different situations. The flow on a stagnation point past a stretching surface was examined by Banks^5^. Khan and Pop^6^ investigated the boundary layer for nanofluid under the impact of Brownian wave theory and concluded that the both phenomena have significant impact on heat flux and the skin friction at the surface. Das et al.^7^ examined the thermal energy influence on the nanofluid. The researchers concentrated on the nanofluid transference by taking base fluid that has invariant fluidic features. The impact of viscosity on the transfer of heat and nanofluid flow was observed by mixing copper and silver nanoparticles that are antimicrobial agents and considered by field emission scanning electron microscope, transmission electron microscope and scanning electron microscope into the water. The experimental results are useful for reproduction filaments^8^. Elbashbeshy^9^ investigated the results of transfer of heat analysis past a stretching sheet. In the recent articles, too much focus was given on the transport of nanofluid and in these articles the base fluid was considered to have invariant rheological characteristics^10^. The influence of variation in viscosity and micro variation on oblige transport of copper–water nanofluid is also examined. Lobatto IIIA method is considered for examining the stability of properties for boundary value problems (BVP). This method is named after Rehuel Lobatto and it is used for the numerical integration of differential equations. Lobatto IIIA method is applied for nonlinear couple system of differential equations arises in the fields of mechanics and electrical circuits^11^.

Generally, industrialized liquids come across having a variable viscosity, which can also be observed as compression, shear or dependent on temperature. Zehra et al.^12^ investigated the flow of non- Newtonian fluid over an inclined channel. The influence of viscosity on drift and warmth transfer with Ag-water and Cu-water nanofluids over a shifting surface is examined by Vajravelu^13^. In recent times, Tabassum et al.^14^ observed the impact temperature is enhanced by increasing the variable viscosity for the nanofluidic system under. Copper steel and silver nanoparticles are frequently used in electronic equipment and thermal conduction^15^. Main functions of copper are in the electrical cable, tiling and pipes used in manufacturing. Numerical work for the flow of Darcy Forchhieimer was studied for the analysis of the sisko nanoparticle by compelling nonlinear thermal radiation with Lobatto IIIA method^16^. The study of Lobatto IIIA was investigated on the porous medium for the flow of two-dimensional flow of magneto hydro dynamic fluid taking the conditions of Navier’s slip and activation energy^17^.

Nanofluid flow pass on a curved surface using Joule heating was considered to study the influence of copper and silver nanoparticles. The nonlinearity in thermal radiation on the curved elongating sheet was examined^18^. The effect of silver and copper nanomaterials on an inclined stretching sheet was investigated to study the effect of viscous fluid with mixed convection on the sheet. Resultant ordinary differential equations are numerically solved by a software Mathematica with Euler’s Explicit Method (EEM)^19^. The effort for the enlargement of heat transfer was analyzed for enlightening the performance of electrical devices by manipulating the porous media for enhancing the heat transfer rate by using different boundary conditions and structures^20^.

Ghadikolaei et al.^21^ investigated the mixed convection flow of hybrid nanoparticles of silver with the 50–50 percent quantity of ethylene–glycol water on the stretching surface in the presence of variable viscosity and different shapes of nanoparticles by a well-known numerical method Runge–Kutta Fehlberg fifth order (RKF-5) and also analyzed the flow of GO-MoS2 hybrid nanoparticles in H2O–(CH2O) hybrid base fluid under the effect of H_2_ bond with (RKF-5) numerical technique^22^. Zangooee^23^ examined the hydrothermal flow of magneto hydro dynamic flow of titanium dioxide with glycol nanofluid flow on the two radiative stretchable revolving disks using AGM. Similarly, many fluidic problems are investigated with numerical and analytical solver such as magnetic field effect on nanofluid flow using AGM and ADM^24–26^, heat transfer rate in nuclear waste^27^, fins arrangement in cubic enclosure^28^, heat transfer simulation in a channel with rectangular cylinder^29,30^ and 3D optimization of baffle arrangement^31^. Besides these, the research community has exploited different numerical schemes in diversified applications^32–39^.

The current article is an attempt to investigate the impact of viscosity in the transfer of heat and thermal conductivity by implementation of numerical scheme based on Lobatto IIIA method. The tabular and diagrammatical illustrations are presented to show the effect of physical quantities for different parameters of the fluidic system^40^. The convergence and stability measures are validated through different tolerances based on residual errors described in the form of tables. The extensive numerical studies and high performance computing^41^ are studies by considering water as base fluid mixing silver and copper nanoparticles under the influence of convection phenomena.

## Model development and description of nanoparticles

**Figure 1:**
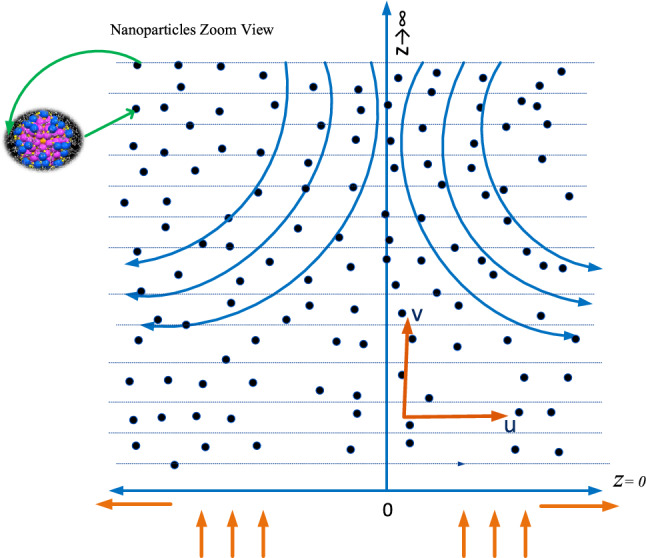
Physical geometry of the proposed problem.

To analyze the effect of nanofluidic system under the impact of heat transfer along a stretching sheet, the rectangular coordinate structure is considered, where x-axis and y-axis are perpendicular and parallel to the stretching sheet in Cartesian coordinates system. The mixed convection with copper and silver in the water is constricted above the surface and stretching sheet is taken along y-axis. ${V}_{w}\left(x\right)=ax,$ where $a$ is the constant, ${V}_{w}\left(x\right)$ is the velocity of a linearly stretching surface and the solid-state two-dimensional stagnation flow is assumed on the surface which is ${V}_{e}\left(x\right)=cy,$ where $c$ is constant. Tabassum^14^ worked on the influence of variation in the resistance. Figure 1 represent the physical geometry of the proposed problem.

Moreover, we supposed the thickness of nanoparticle in the Vogel’s model. In the direction of y-axis, a uniform magnetic field ${B}_{o}$ is applied in the normal direction of the stretching surface. The uniform magnetic field is induced due to magnetic Reynolds number which is taken very small that can be neglected as compared to magnetic field. Maraj^24^ express the system of governing equations in the following form:

1 $$ \frac{{\partial \overline{u} }}{\partial x} + \frac{{\partial \overline{v} }}{\partial y} = 0, $$

2 $$ \overline{u} \frac{{\partial \overline{u} }}{\partial x} + \overline{v} \frac{{\partial \overline{u} }}{\partial y} = V_{e} \frac{{dV_{e} }}{dx} + \frac{1}{{\uprho _{nf} }}\frac{\partial }{\partial y}\left( {\upmu _{nf} (T)\frac{{\partial \overline{u} }}{\partial y}} \right) + \frac{{g\upbeta }}{{\uprho _{nf} }}(T - T_{\infty } ) - \frac{{\upsigma _{nf} }}{{\uprho _{nf} }}B_{o}^{2} (\overline{u} - V_{e} ), $$

3 $$ \overline{u} \frac{\partial T}{{\partial x}} + \overline{v} \frac{\partial T}{{\partial y}} = \frac{{k_{nf} }}{{(\uprho cp)_{nf} }}\frac{{\partial^{2} T}}{{\partial y^{2} }}. $$

where, $T_{z}$ and $T_{\infty }$ are temperatures at the surface and away from the surface.

The Boundary conditions are

4 $$ \begin{aligned} & \overline{u} - V_{w} = cx,\;\overline{v} = 0,\;T = T_{z} \;{\text{at}}\;y = 0, \\ & \overline{u} \to V_{e} = ax,\;T \to T_{\infty } \;{\text{at}}\;y \to \infty . \\ \end{aligned} $$

where $\overline{u}$ and $\overline{v}$ are components of velocity along $x$ and $y$ direction, respectively $\upsigma _{nf} ,k_{nf} ,\uprho _{nf,}\upmu _{nf}$ and $(cp)_{nf}$ are electrical conductivity, thermal conductivity representing the rate at with heat permits through the fluid, density, dynamic viscosity and specific heat capacity of nanofluid. The properties of base fluid and nanoparticles is represented in Table 1. Table 2 represents the thermophysical properties of $\upsigma _{nf} ,k_{nf} ,\upmu _{nf}$,$\rho_{nf}$.

**Table 1:** The physical properties of nanoparticles and base fluids and their Investigational values.

Properties	Water	Cu	Ag
Density, $\uprho$(kg/$m^{3}$)	997	8933	10,500
Thermal conductivity, k	0.613	400	429
Specific heat,$C_{p}$	4179	385	235

**Table 2:** The physical expressions of nanofluids.

Properties	Nanofluids
Density	$\rho_{nf} = \rho_{f} \left( {\left( {1 - \varphi } \right) + \varphi \left( {\frac{{\rho_{s} }}{{\rho_{f} }}} \right)} \right)$
Viscosity	$\mu_{nf} = \frac{{\mu_{f} }}{{\left( {1 - \varphi } \right)^{2.5} }}$
Thermal conductivity	$\frac{{k_{nf} }}{{k_{f} }} = \frac{{k_{s} + (m - 1)k_{f} - \varphi (m - 1)(k_{f} - k_{s} )}}{{k_{s} + k_{f} (m - 1) + \varphi (k_{f} - k_{s} )}}$
Heat capacity	$(\rho C_{p} )_{nf} = (\rho C_{p} )_{f} \left( {1 - \varphi + \varphi \left( {\frac{{(\rho C_{p} )_{s} }}{{(\rho C_{p} )_{f} }}} \right)} \right)$

The variable viscosity measures the resistance occurs due to deformation of the fluid represented in the form of Vogel’s model as

5 $$ \mu_{nf} = \mu_{f} \left( {1 - \beta \left( {T - T_{\infty } } \right)} \right) $$

Using the following conversions

6 $$\upeta = y\sqrt {\frac{c}{{v_{f} }}} ,\overline{u} = cxf^{{\prime }} (\upeta ),y = - \sqrt {cv_{f} } f(\upeta ),\theta (\upeta ) = \frac{{T - T_{\infty } }}{{T_{z} - T_{\infty } }}. $$

Equation (1) is correctly satisfied and Eq. (2) for Vogel’s model and energy Eq. (3) takes the form as

7 $$ f^{\prime^2} - ff^{\prime \prime } - Gr\theta + M^{2} f^{\prime } + B_{1}\uplambda f^{\prime }\theta^{\prime } - A\left( {A - M^{2} } \right) = 0 $$

8 $$ \theta^{\prime \prime } + \Pr B_{3} B_{4} f\theta^{\prime } = 0 $$

where $B_{i} \left( {i = 1,2,3,4} \right)$ are defined as

$$ B_{1} = \left( {1 - {\varphi }} \right)^{2.5} $$

9 $$ B_{2} = \left( {1 - {\varphi }} \right) + {\varphi }\left( {\frac{{\uprho _{s} }}{{\uprho _{f} }}} \right) $$

$$ B_{3} = \frac{{k_{s} + \left( {m - 1} \right)k_{f} + {\varphi }\left( {k_{f} - k_{s} } \right)}}{{k_{s} + \left( {m - 1} \right)k_{f} - \left( {m - 1} \right){\varphi }\left( {k_{f} - k_{s} } \right)}} $$

10 $$ B_{4} = \left( {1 - {\varphi }} \right) + {\varphi }\frac{{\left( {\uprho c_{p} } \right)_{s} }}{{\left( {\uprho c_{p} } \right)_{f} }} $$

The boundary conditions are

11 $$ \begin{gathered} f\left(\upeta \right) = 0,\;f^{\prime }\left(\upeta \right) = 1,\;\theta \left(\upeta \right) = 1\;{\text{at}}\;\upeta = 0, \hfill \\ f^{\prime }\left(\upeta \right) \to A,\;\theta \left(\upeta \right) \to 0\;{\text{as}}\;\upeta = \infty , \hfill \\ \end{gathered} $$

Mathematical expressions for Grashof number *Gr*, Hartmann number *M*, stretching ratio parameter *A*, Prandalt number *Pr* and viscosity parameter $\uplambda$ which are used in above equations are

12 $$ Gr = \frac{{g\upbeta }}{{\uprho _{f} c^{2} y}}\left( {T_{w} - T_{\infty } } \right),\;M = \frac{{\upsigma _{nf} B^{2}_{0} }}{{\uprho _{f} c}},\;A = \frac{a}{c},\;\Pr = \frac{{k_{f} }}{{\left( {\uprho c_{p} } \right)_{f} }},\;\uplambda =\upbeta \left( {T_{z} - T_{\infty } } \right)C_{p} . $$

The physical quantities alike rate of heat flux $Nu{}_{x}$ and shear strain $Cf_{x}$ and at the surface can be expressed as

13 $$ Nu_{x} = \frac{{xq_{z} }}{{k_{f} \left( {T_{z} - T_{\infty } } \right)}},\;Cf_{x} = \frac{{\tau_{z} }}{{\rho \left( {u_{z} } \right)^{2} }} $$

where, $\tau_{z}$ represent the shear stress of wall and $q_{z}$ is heat flux at wall. The dimensionless form of above expression by invoking similarity transformation is

14 $$ \frac{{Nu_{x} }}{{\sqrt {{\text{Re}}_{x} } }} = \frac{{k_{nf} }}{{k_{f} }}\theta^{\prime\prime}\left( 0 \right),\;\sqrt {{\text{Re}}_{x} } Cf_{x} = \frac{1}{{\left( {1 - \varphi } \right)^{2.5} }}f^{\prime\prime}\left( 0 \right), $$

where, the term $\sqrt {{\text{Re}}_{x} } = \frac{{u_{z} x}}{{v_{f} }}$ is local Reynolds’s number.

## Solution methodology

**Figure 2:**
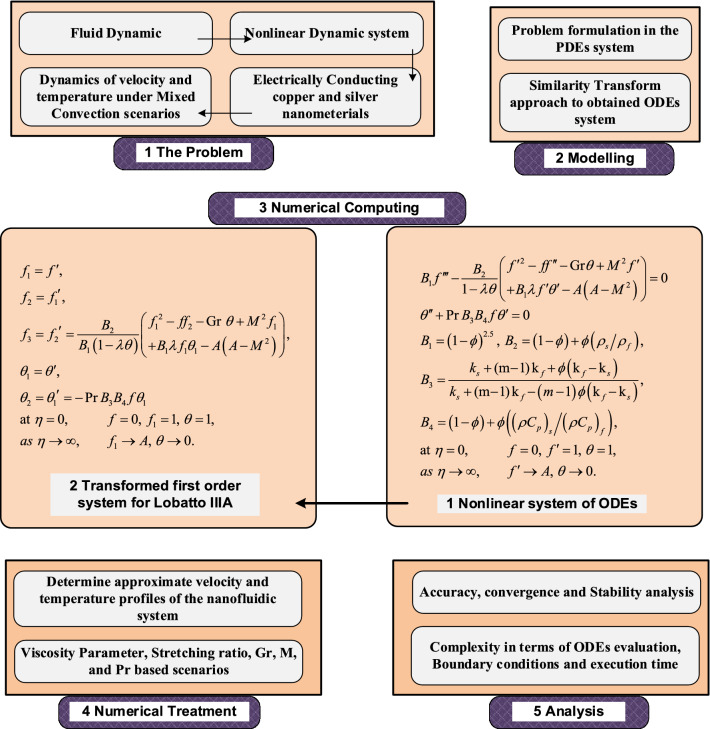
Graphical abstract of proposed problem.

To find the solution of coupled nonlinear Eqs. (7–8) with the boundary conditions in Eq. (12). Lobatto IIIA is incorporated with MATLAB routine ‘bvp4c’. The strategy for the solution of problem is presented in Fig. 2. The first step in the diagram provides the fluid flow system dynamics, the transformation system for PDEs to ODEs is given in the second step, the third step is consisting of two parts i.e., non-linear higher orders ODEs of the fluidic system and their conversion into first order system of ODEs. In the fourth step of the fluid flow diagram, velocity and temperature profiles are attained based on parameters of interest of the flow system, while the last step in the block diagram represents the accuracy, convergence, and stability analysis in terms of residual errors.

## Physical and tabulated description of results

**Figure 3:**
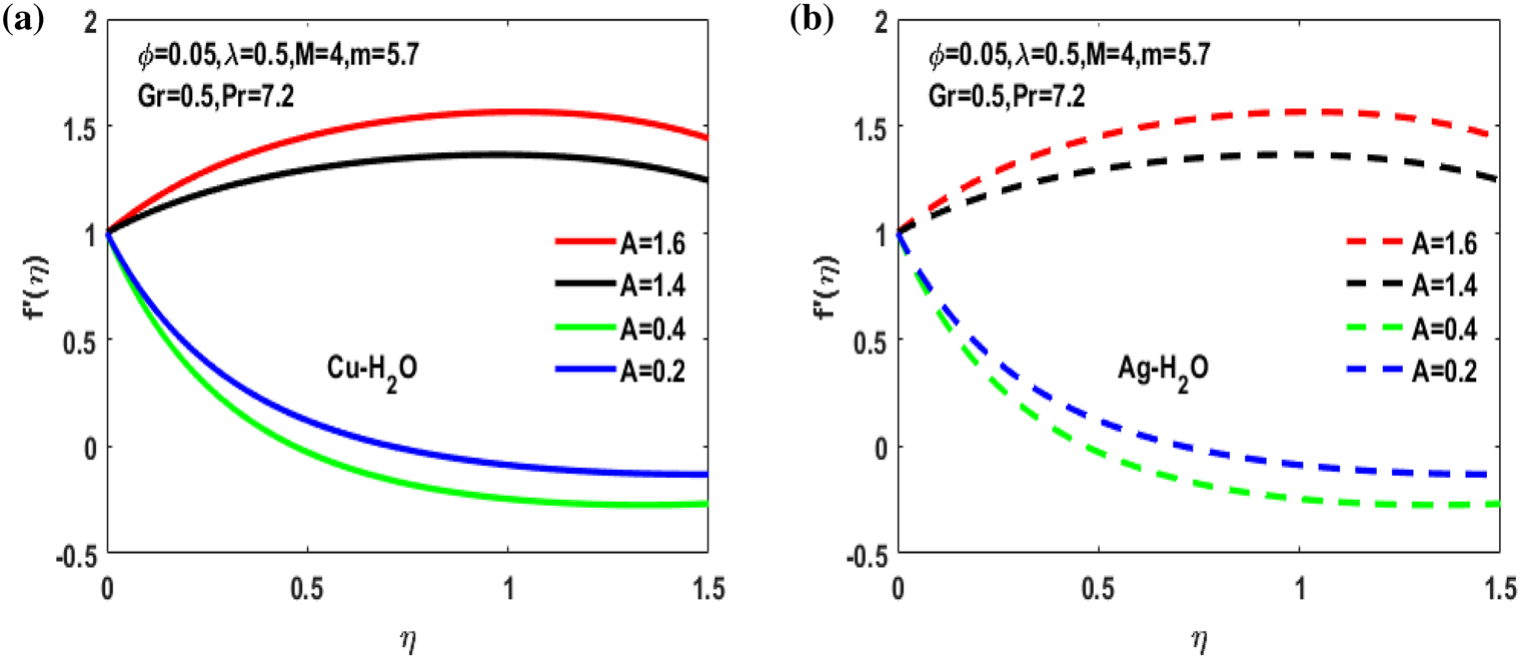
(**a**) Variation of A for ${f}^{^{\prime}}\left(\eta \right)$. (**b**) Variation of A for ${f}^{^{\prime}}\left(\eta \right).$

**Figure 4:**
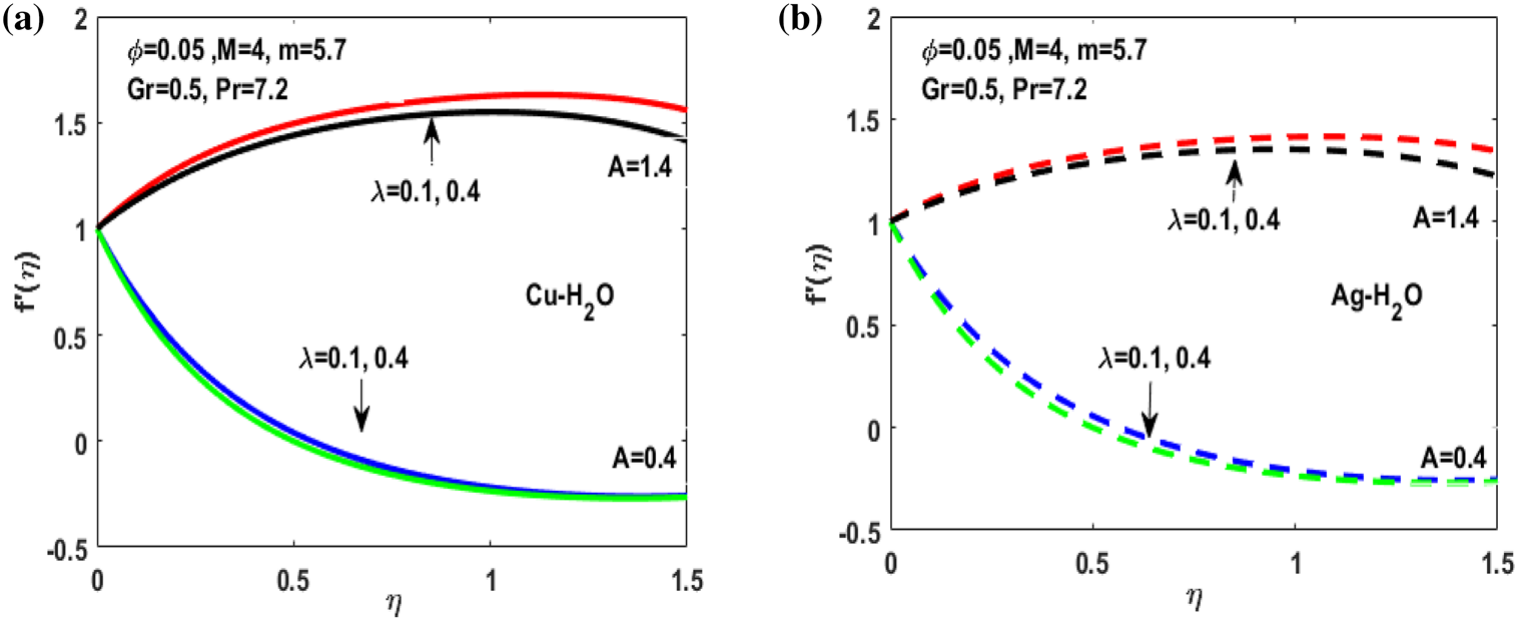
(**a**) Variation of $\lambda$ for ${f}^{^{\prime}}\left(\eta \right)$ **.** (**b**) Variation of $\lambda$ for ${f}^{^{\prime}}\left(\eta \right).$

**Figure 5:**
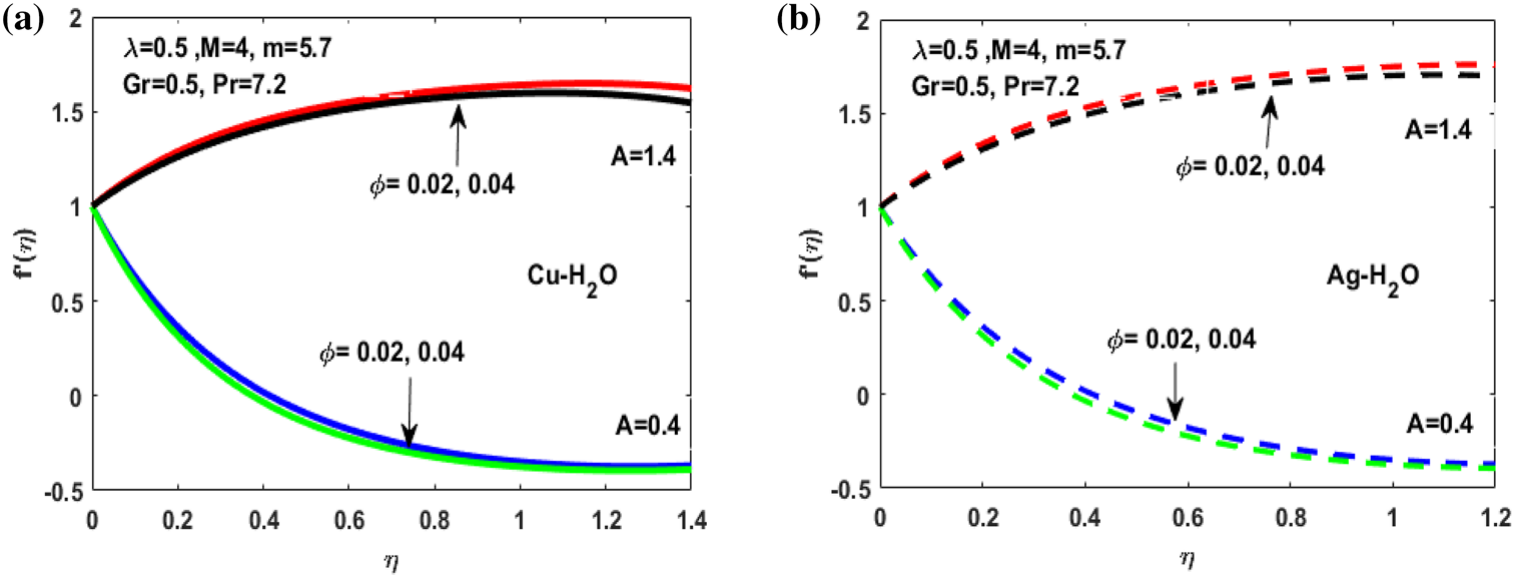
(**a**) Variation of $\Phi$ for ${f}^{^{\prime}}\left(\eta \right)$**.** (**b**) Variation of $\Phi$ on ${f}^{^{\prime}}\left(\eta \right).$

**Figure 6:**
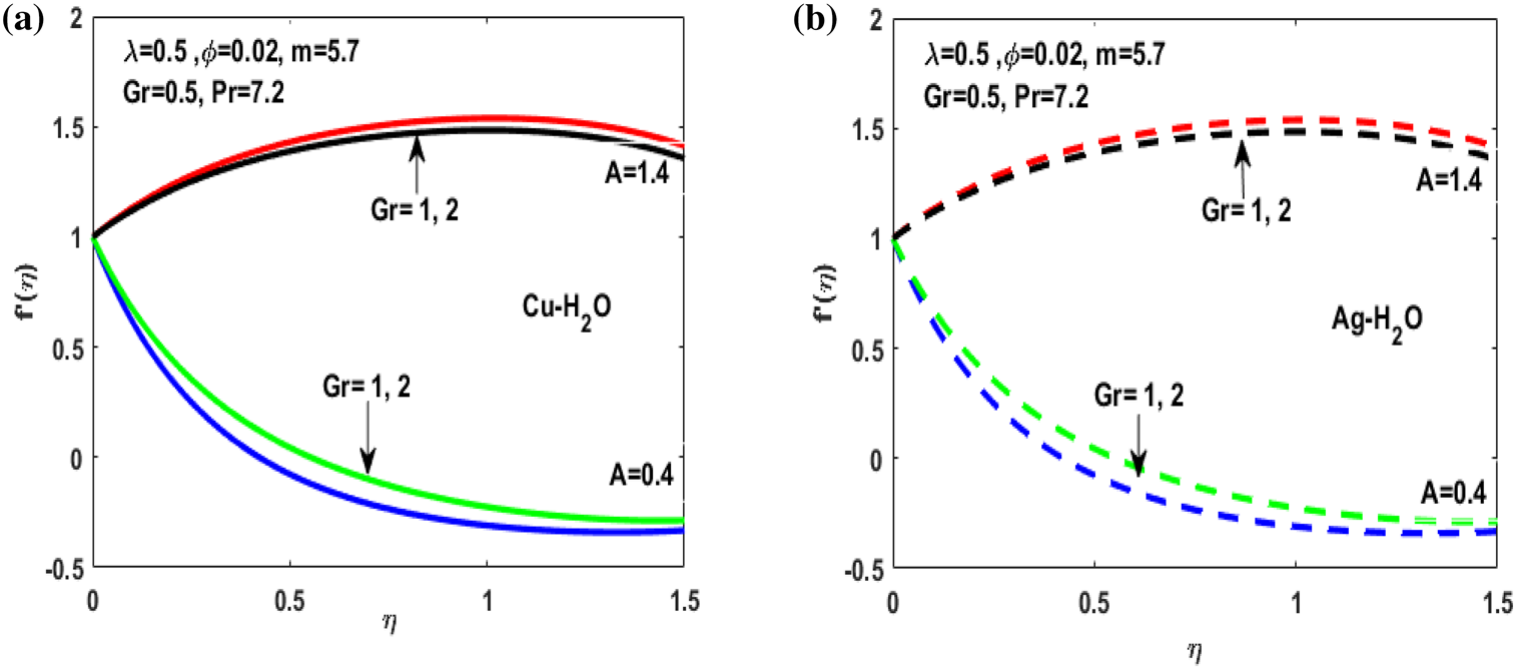
(**a**) Impact of Gr on ${f}^{^{\prime}}\left(\eta \right)$. (**b**) Impact of Gr on ${f}^{^{\prime}}\left(\eta \right).$

**Figure 7:**
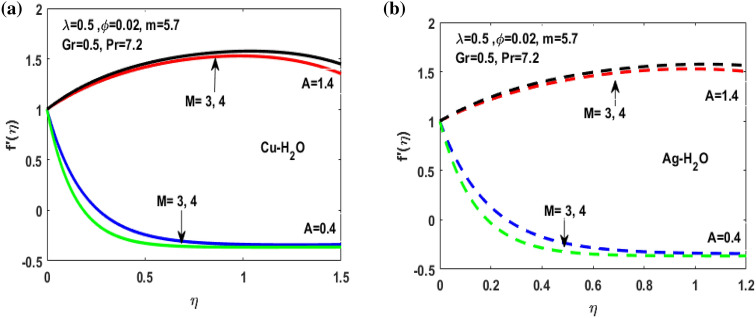
(**a**) Variation of M for ${f}^{^{\prime}}\left(\eta \right)$**.** (**b**) Variation of M for ${f}^{^{\prime}}\left(\eta \right).$

**Figure 8:**
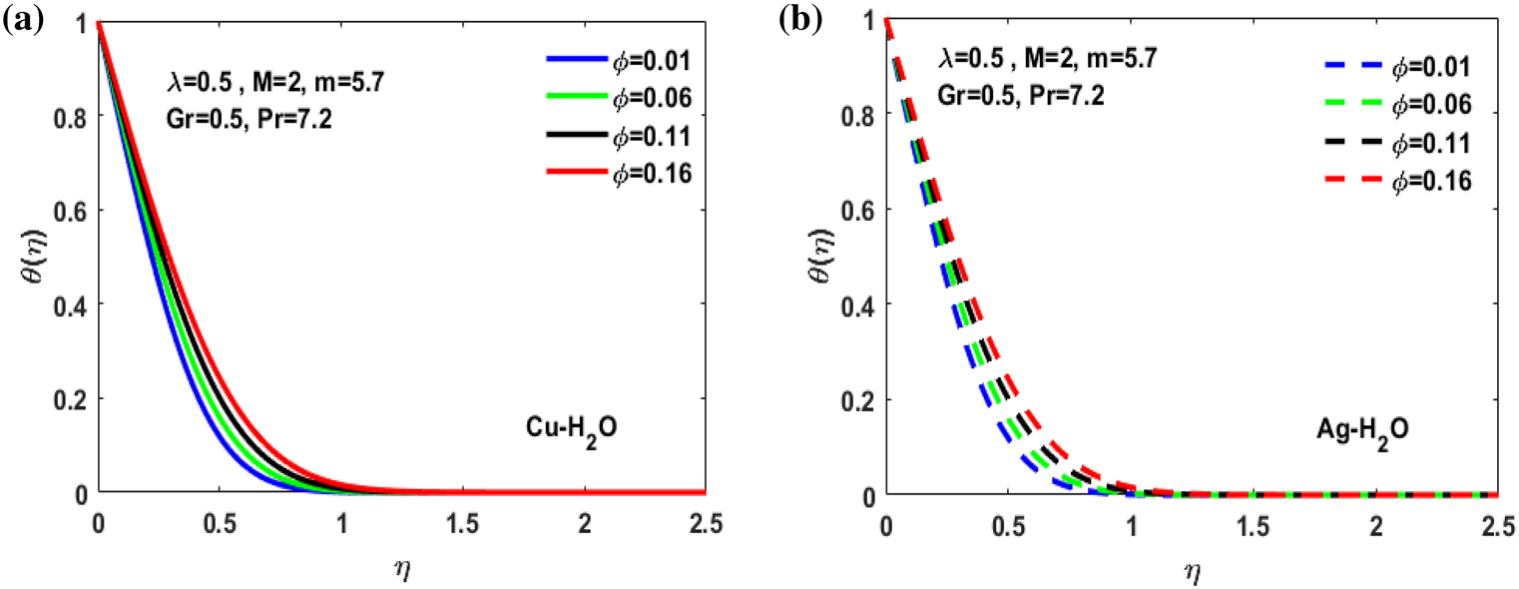
(**a**) Effect of $\Phi$ on $\theta (\eta )$*.* (**b**) Effect of $\Phi$ on $\theta (\eta )$*.*

**Figure 9:**
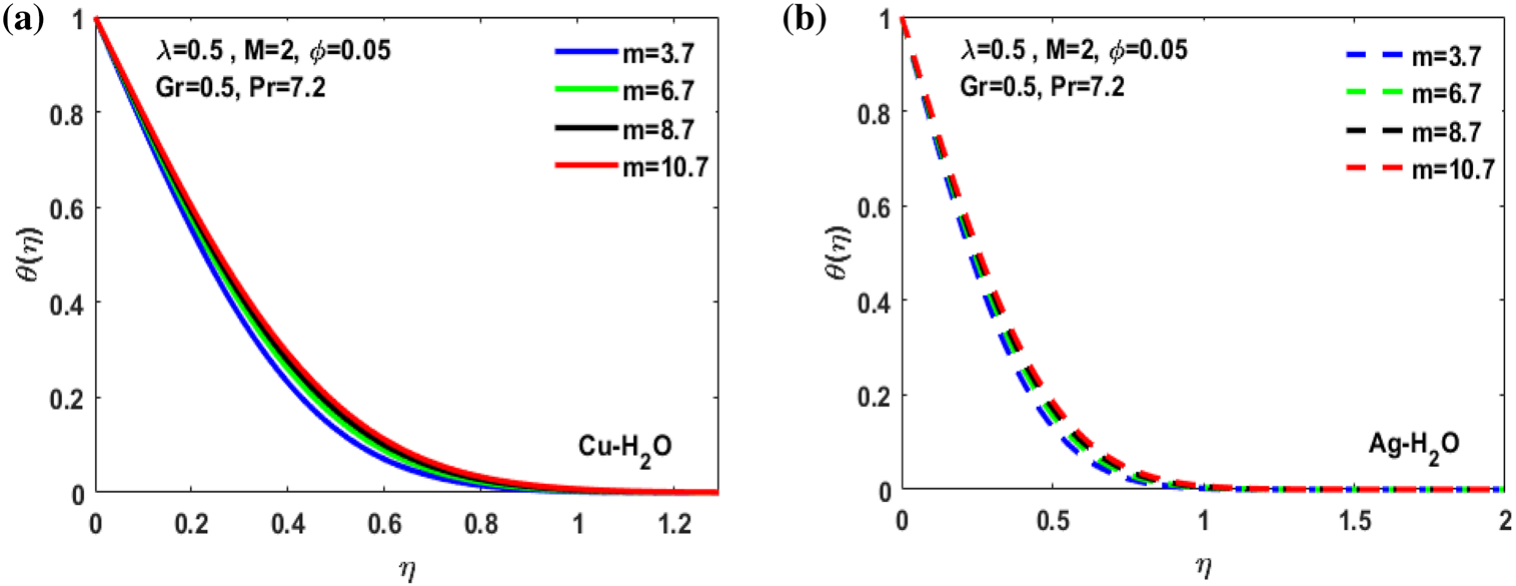
(**a**) Inspiration of m on $\theta$($\eta$). (**b**) Inspiration of m on $\theta$($\eta$).

**Figure 10:**
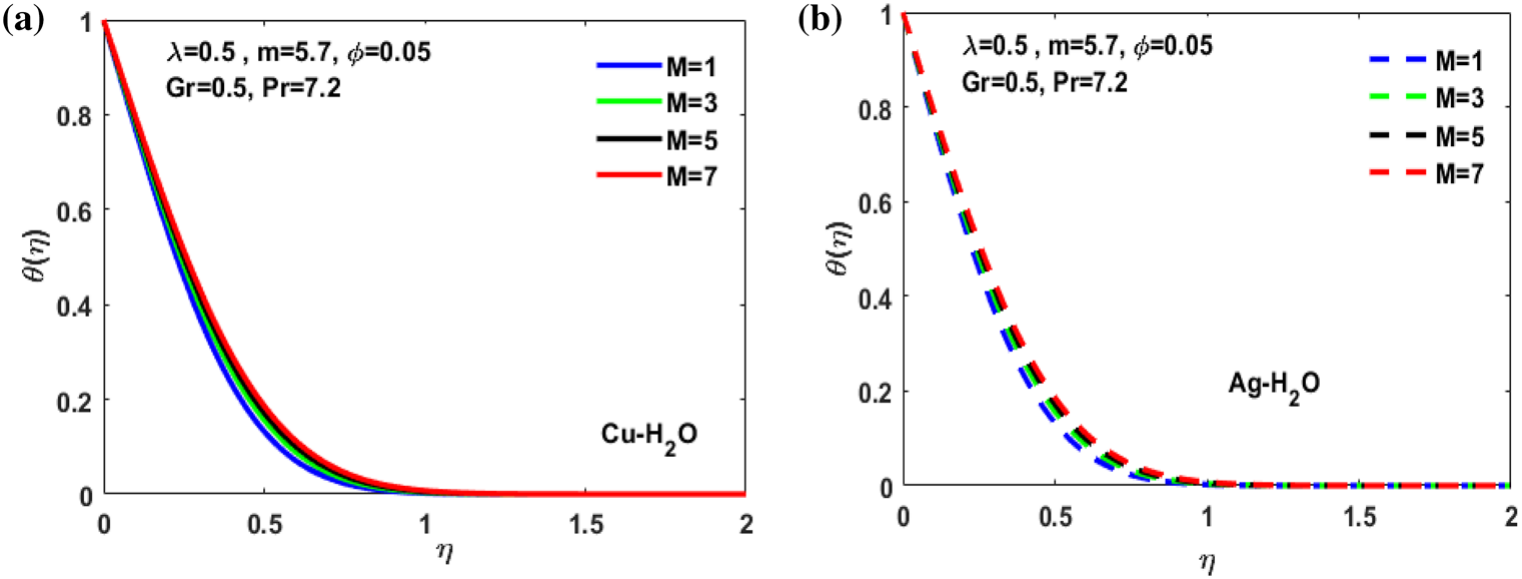
(**a**) Variation of $M$ on $\theta \left(\eta \right)$**.** (**b**) Variation of $M$ on $\theta \left(\eta \right).$

**Figure 11:**
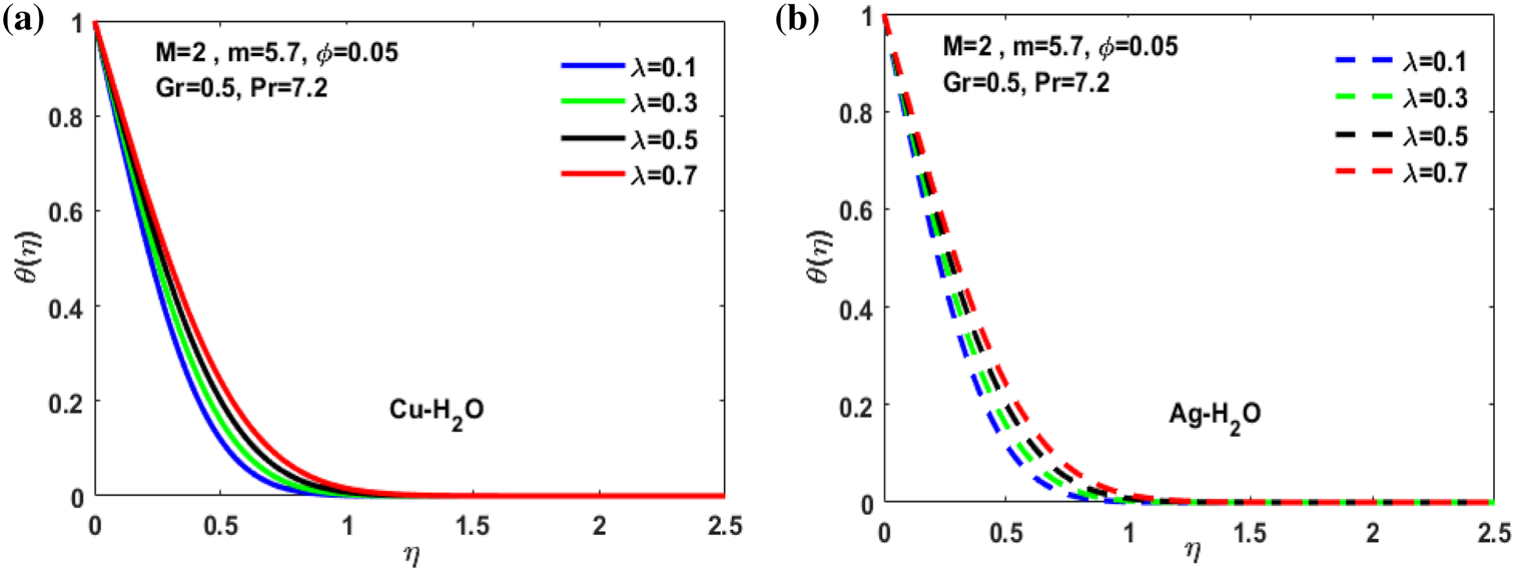
(**a**) Inspiration of $\lambda$ on $\theta \left(\eta \right)$**.** (**b**) Inspiration of $\lambda$ on $\theta \left(\eta \right).$

**Figure 12:**
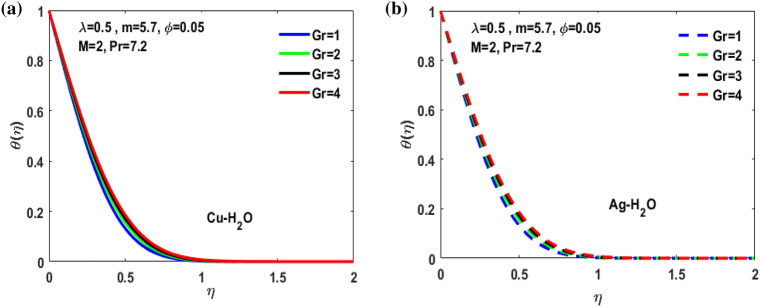
(**a**) Impact of Gr on $\theta \left(\eta \right)$**.** (**b**) Impact of Gr on $\theta \left(\eta \right).$

The nonlinear ordinary differential equations are solved with Lobatto IIIA method by using MATLAB software. The numerical solution obtained for the distribution of temperature and velocity are observed and showed through diagrams. Figures 3, 4, 5, 6, 7, 8, 9, 10, 11 and 12 shows the consequence of extending ratio parameter, volumetric fraction, magnetic parameter, Grashof number and viscosity parameter for Ag-Water and Cu-Water for the Vogel’s model. Here we discuss the results for both A < 1 and A > 1.

Figures 3, 4, 5, 6 and 7 shows the impact of extending ratio parameter A in the viscosity model of temperature dependent it is observed that the viscosity is concave up and concave down for both the increasing and decreasing value of elongating ratio parameter. The solid lines represent the viscosity profile for copper–water nanofluid, and dashed lines represent the viscosity profile for silver-water nanofluid.

Figure 3a,b it is examined that when A < 1 the velocity profile is concave down for variation in ratio parameter A for both silver and copper particles with water based nanofluids and velocity profile is concave up for the increasing value of elongating ratio parameter. It is observed that the decreasing value of A represent the condition when stagnation velocity is less than elongating ratio and the increasing value of A represent the state when the stagnation velocity becomes greater than the ratio parameter A. Figure 4a,b shows that the viscosity parameter decreases for A < 1 and goes on increasing when A > 1. It is observed that the velocity decreases with increasing value of viscosity parameter. In Fig. 5a,b behavior of volume fraction for Cu and Ag is shown. It shows that the flow accelerates for increasing value of volume fraction when the value of ratio parameter is decreasing, and the flow behaves oppositely when the value of elongating ratio parameter is increasing. Figure 6a,b shows the result of Grashof number and it is observed that the velocity increase for increasing the values of Grashof number. The velocity increases because Grashof number is proportion of resistance forces to the viscid forces. Figure 7a,b represent the impact of magnetic field and it shows that the magnetic field constantly turns as a resistive energy so the velocity decreases for increasing values of magnetic field for both silver and copper based nanofluids. Figures 8, 9, 10, 11 and 12 shows the effect of temperature for different values of parameters such as nanoparticle shape m, Grashof number Gr, magnetic field M, viscosity parameter $\uplambda$ and viscosity parameter ${\varphi }$ for both the nanoparticles i.e. copper and silver. In Fig. 8a,b it is observed that the presence of nanoparticle enhance the base fluid capacity to bearing heat and therefore, temperature droplets so that is why due to high volumetric fraction ${\varphi }$ the temperature of the fluid is declined. Figure 9a,b shows that for increasing the value of particle shape factor m temperature decreases and maximum temperature is illustrious for blade formed nanoparticle whereas minimum temperature is observed for bricked formed particles. Figure 10a,b shows the influence of magnetic parameter M. It is observed that in the presence of external magnetic field temperature rises. Impact of fluid viscosity on temperature is represented in Fig. 11a,b. It is observed that temperature of fluidic syste upsurges due to dependence of viscosity on temperature. Figure 12a,b is plotted for different values of Grashof number and the mixed convection (as a result of combining both natural and force convection phenomena) cause to rise in temperature for both silver and copper nanofluid. The Grashof number arises in the study of the situations develops due to natural convection heat transfer phenomena and it approximates the relation between buoyancy and viscous forces exerted on the fluidic flow system under the impact of variable viscosity and mixed convection. The temperature of the fluid accelerates due the blade type nanoparticles of copper and skin friction coefficient is reduced due to enhancement of Grashof Number. Tabulated results for four different convergence limits for checking the reliability of solution are shown in Tables 3, 4, 5 and 6 for both the silver and copper nanoparticles. Tables 7, 8 shows the skin friction and Nusselt number coefficient for different scenario and cases for both the copper–water and silver-water nanofluids. The complexity of existing system is analyzed through the number of ODEs and BCs to attain desirable residual error in Table 3 (a)-(b). In this tables different values are assigned to sundry variables in various cases. There are 10 scenarios and 4 cases for each scenario, which shows variation of each parameter. Table 4 (a)-(b) represents number of nmesh points for each case of different scenarios. Table 5 (a)-(b) represent number of ODEs evaluated for the solution. Table 6 (a)-(b) represent number of boundary conditions evaluations associated with the fluid flow system. Table 7 (a)-(b) shows Nusselt number for both copper and silver nanoparticles and Table 8 (a)_(b) shows the skin friction for different scenarios and cases.

**Table 3:** Maximum Residual during different scenario and cases for (a) Copper nanoparticles, (b) Silver nanoparticles.

Convergence limit	Scenario	Case 1	Case 2	Case 3	Case 4
**(a)**					
1e−06	1	9.5394e−09	6.5803e−09	3.1793e−08	3.2353e−08
	2	1.3195e−08	4.4664e−08	5.7417e−09	1.6598e−08
	3	1.0632e−07	7.0759e−09	4.9350e−08	2.2043e−08
	4	6.1913e−08	1.1706e−07	3.0572e−08	4.3764e−08
	5	2.0373e−07	3.5570e−08	1.1108e−08	6.8914e−09
1e−08	1	2.0253e−10	1.1368e−10	4.8106e−09	4.8647e−09
	2	1.2398e−09	2.2764e−09	1.1642e−09	4.4560e−10
	3	2.0334e−09	9.7852e−11	2.2934e−09	7.3143e−10
	4	4.9843e−09	1.3356e−09	1.6415e−09	3.6626e−09
	5	2.1496e−09	1.1164e−09	2.7152e−10	1.6171e−10
1e−10	1	2.0253e−12	1.1368e−12	4.8106e−11	4.8647e−11
	2	1.2398e−11	2.2764e−11	1.1642e−11	4.4560e−12
	3	2.0334e−11	9.7852e−13	2.9350e−11	7.6807e−12
	4	4.9843e−11	1.3356e−11	1.9569e−11	4.7243e−11
	5	2.1496e−11	1.1164e−11	2.7152e−12	1.6171e−12
1e−12	1	2.0253e−14	1.1368e−14	4.8106e−13	4.8647e−13
	2	1.2398e−13	2.2764e−13	1.1642e−13	4.4560e−14
	3	2.0334e−13	9.7852e−15	2.9350e−13	7.6807e−14
	4	4.9843e−13	1.3356e−13	1.9569e−13	4.7243e−13
	5	2.1496e−13	1.1164e−13	2.7152e−14	1.6171e−14
**(b)**					
1e−06	1	7.6701e−09	5.5001e−09	3.4347e−08	9.1614e−07
	2	1.4144e−08	4.8001e−08	4.9094e−09	1.2862e−08
	3	1.5838e−07	7.4915e−09	4.7962e−08	1.9254e−08
	4	6.7115e−08	1.2696e−07	2.7468e−08	4.2121e−08
	5	2.6675e−07	2.1128e−07	8.6183e−09	5.2881e−09
1e−08	1	1.3755e−10	8.2593e−11	1.6009e−09	4.9189e−09
	2	1.4083e−09	4.1337e−09	8.6046e−10	2.9163e−10
	3	2.8454e−09	1.0840e−10	2.1582e−09	5.7196e−10
	4	5.7006e−09	1.5291e−09	1.3104e−09	3.1458e−09
	5	2.9857e−09	2.1157e−09	1.7879e−10	1.1253e−10
1e−10	1	1.3755e−12	8.2593e−13	1.6009e−11	4.9189e−11
	2	1.4083e−11	4.1337e−11	8.6046e−12	2.9163e−12
	3	2.8454e−11	1.0840e−12	2.7637e−11	5.8787e−12
	4	5.7006e−11	1.5291e−11	1.5543e−11	4.2255e−11
	5	2.9857e−11	2.1157e−11	1.7879e−12	1.1253e−12
1e−12	1	1.3755e−14	8.2593e−15	1.6009e−13	4.9189e−13
	2	1.4083e−13	4.1337e−13	8.6046e−14	2.9163e−14
	3	2.8454e−13	1.0840e−14	2.7637e−13	5.8787e−14
	4	5.7006e−13	1.5291e−13	1.5543e−13	4.2255e−13
	5	2.9857e−13	2.1157e−13	1.7879e−14	1.1253e−14

**Table 4:** Number of Mesh points during different scenario/cases for (a) Copper nanoparticles, (b) Silver nanoparticles.

Convergence limit	Scenario	Case 1	Case 2	Case 3	Case 4
**(a)**					
1e−06	1	1297	1272	909	1323
	2	793	1018	1469	1429
	3	600	1221	1576	1473
	4	1440	1590	1572	1623
	5	600	1164	1320	1339
1e−08	1	1368	1350	935	1380
	2	795	1100	1508	1487
	3	600	1282	1602	1525
	4	1622	1713	1594	1646
	5	600	1193	1382	1411
1e−10	1	1368	1350	935	1380
	2	795	1100	1508	1487
	3	600	1282	1602	1525
	4	1622	1713	1594	1646
	5	600	1193	1382	1411
1e−12	1	1368	1350	935	1380
	2	795	1100	1508	1487
	3	600	1282	1602	1525
	4	1622	1713	1594	1646
	5	600	1193	1382	1411
**(b)**					
1e−06	1	1278	1248	1021	600
	2	789	992	1463	1335
	3	600	1240	1573	1433
	4	1452	1585	1552	1619
	5	600	600	1311	1323
1e−08	1	1352	1328	1086	604
	2	791	1065	1503	1397
	3	600	1292	1598	1487
	4	1634	1715	1578	1634
	5	600	600	1379	1404
1e−10	1	1352	1328	1086	604
	2	791	1065	1503	1397
	3	600	1292	1598	1487
	4	1634	1715	1578	1634
	5	600	600	1379	1404
1e−12	1	1352	1328	1086	604
	2	791	1065	1503	1397
	3	600	1292	1598	1487
	4	1634	1715	1578	1634
	5	600	600	1379	1404

**Table 5:** Number of ODEs assessed for variants (a) Cu nanoparticles, (b) Silver nanoparticles.

Convergence limit	Scenario	Case 1	Case 2	Case 3	Case 4
**(a)**					
1e−06	1	38,457	38,130	26,797	30,994
	2	22,517	27,995	37,691	40,173
	3	16,797	29,668	42,084	40,745
	4	35,517	37,467	42,032	42,695
	5	16,201	28,926	38,756	39,003
1e−08	1	39,380	39,144	28,952	34,494
	2	24,128	31,096	38,198	40,927
	3	16,797	30,461	42,422	41,421
	4	37,883	42,491	42,318	46,285
	5	16,201	29,303	39,562	39,939
1e−10	1	39,380	39,144	28,952	34,494
	2	24,128	31,096	41,213	43,900
	3	16,797	33,024	45,625	44,470
	4	41,126	42,491	45,505	46,285
	5	16,201	31,688	42,325	42,760
1e−12	1	42,115	41,843	28,952	37,253
	2	24,128	31,096	41,213	43,900
	3	16,797	33,024	45,625	44,470
	4	44,369	45,916	45,505	49,576
	5	16,201	31,688	42,325	42,760
**(b)**					
1e−06	1	38,210	37,818	28,028	16,800
	2	22,473	27,708	37,613	38,951
	3	16,798	29,915	42,045	40,225
	4	35,673	37,400	41,772	42,643
	5	16,800	16,799	38,639	38,795
1e−08	1	39,172	38,858	30,914	23,442
	2	24,076	30,640	38,133	39,757
	3	16,798	30,591	42,370	40,927
	4	38,039	42,519	42,110	46,105
	5	16,800	16,799	39,523	39,848
1e−10	1	39,172	38,858	30,914	24,649
	2	24,076	30,640	41,138	42,550
	3	16,798	33,174	45,565	43,900
	4	41,306	42,519	45,265	46,105
	5	16,800	16,799	42,280	42,655
1e−12	1	41,875	41,513	33,085	24,649
	2	24,076	30,640	41,138	42,550
	3	16,798	33,174	45,565	43,900
	4	44,573	45,948	45,265	49,372
	5	16,800	16,799	42,280	42,655

**Table 6:** Number of BCs assessed for variants of (a) Copper nanoparticles, (a) Silver nanoparticles.

Convergence limit	Scenario	Case 1	Case 2	Case 3	Case 4
**(a)**					
1e−06	1	93	93	75	59
	2	58	74	77	93
	3	56	59	93	93
	4	75	75	93	93
	5	56	59	93	93
1e−08	1	93	93	76	60
	2	59	75	77	93
	3	56	59	93	93
	4	75	76	93	94
	5	56	59	93	93
1e−10	1	93	93	76	60
	2	59	75	78	94
	3	56	60	94	94
	4	76	76	94	94
	5	56	60	94	94
1e−12	1	94	94	76	61
	2	59	75	78	94
	3	56	60	94	94
	4	77	77	94	95
	5	56	60	94	94
**(b)**					
1e−06	1	93	93	75	56
	2	58	74	77	93
	3	56	59	93	93
	4	75	75	93	93
	5	56	56	93	93
1e−08	1	93	93	76	74
	2	59	75	77	93
	3	56	59	93	93
	4	75	76	93	94
	5	56	56	93	93
1e−10	1	93	93	76	75
	2	59	75	78	94
	3	56	60	94	94
	4	76	76	94	94
	5	56	56	94	94
1e−12	1	94	94	77	75
	2	59	75	78	94
	3	56	60	94	94
	4	77	77	94	95
	5	56	56	94	94

**Table 7:** Nusselt numbers evaluated during different scenario and cases for (a) Copper nanoparticles, (b) Silver nanoparticles.

Scenarios	Case-1	Case-2	Case-3	Case-4
**(a)**				
S1	0.430515	0.100593	− 5.86467	− 5.31517
S2	− 4.04844	− 5.93804	1.071699	0.718761
S3	− 4.58793	− 5.07748	1.163714	0.92052
S4	− 8.60141	− 11.7084	1.251741	1.631453
S5	− 4.84596	− 4.06429	0.646338	0.379216
S6	3.226849	3.560967	3.721918	3.879947
S7	3.880285	3.880117	3.879947	3.879025
S8	3.190446	3.919002	5.427194	7.170683
S9	2.415066	2.818688	3.396271	4.355369
S10	3.55902	3.882205	4.202432	4.51984
**(b)**				
S1	0.331157	0.034014	− 6.02992	− 5.47097
S2	− 4.16066	− 6.1106	0.939037	0.58602
S3	− 4.65941	− 5.20655	1.128166	0.828158
S4	− 8.83948	− 12.0327	1.131419	1.509756
S5	− 4.98917	− 4.1758	0.523754	0.278394
S6	3.25434	3.636215	3.818451	3.996481
S7	3.997616	3.996962	3.996481	3.994511
S8	3.250983	3.976884	5.505487	7.272729
S9	2.447695	2.859302	3.448647	4.42806
S10	3.61551	3.946837	4.275094	4.600427

**Table 8:** Skin friction evaluated during different scenario and cases for (a) Copper nanoparticles, (b) Silver nanoparticles.

Scenarios	Case-1	Case-2	Case-3	Case-4
**(a)**				
S1	− 0.01316	− 0.00308	0.179321	0.162519
S2	0.123787	0.181564	− 0.03277	− 0.02198
S3	0.140283	0.155252	− 0.03558	− 0.02815
S4	0.263001	0.358002	− 0.03827	− 0.04988
S5	0.148172	0.124272	− 0.01976	− 0.0116
S6	− 0.09867	− 0.10888	− 0.1138	− 0.11864
S7	− 0.11865	− 0.11864	− 0.11864	− 0.11861
S8	− 0.09755	− 0.11983	− 0.16594	− 0.21925
S9	− 0.07384	− 0.08619	− 0.10385	− 0.13317
S10	− 0.10882	− 0.1187	− 0.1285	− 0.1382
**(b)**				
S1	− 0.01013	− 0.00104	0.184374	0.167283
S2	0.127219	0.186841	− 0.02871	− 0.01792
S3	0.142468	0.159198	− 0.0345	− 0.02532
S4	0.27028	0.367917	− 0.03459	− 0.04616
S5	0.152551	0.127681	− 0.01601	− 0.00851
S6	− 0.09951	− 0.11118	− 0.11675	− 0.1222
S7	− 0.12223	− 0.12221	− 0.1222	− 0.12214
S8	− 0.0994	− 0.1216	− 0.16834	− 0.22237
S9	− 0.07484	− 0.08743	− 0.10545	− 0.13539
S10	− 0.11055	− 0.12068	− 0.13072	− 0.14066

## Conclusions

In the present article, we discussed the results of mixed convection on MHD nanofluid and temperature dependent viscosity of the fluid by incorporating the strength of numerical computational approach. In this flow the stagnation point was towards the extending sheet. Copper-silver nanoparticles with base fluid were used for complete analysis. Vogel’s model was taken to determine the impact of viscosity which is depending on temperature. Problem was expressed in the x–y coordinate system. The results of present analysis give the following key findings:

- The fluid flow is decreased due to temperature dependent viscosity.
- The fluid flow of assorted convection is affected by accelerating it.
- The rise in volumetric fraction of nanoparticles causes to increase the thermal conductivity of the traditional fluid.
- The impact of temperature was maximum for blade type nanoparticles of copper.
- Skin friction coefficient decreases with the increasing value of Grashof Number *Gr* and increased with the increasing value of viscosity factors and volume fraction while these parameters reduced the Nusselt Number.

In future, one may implement the Lobatto IIIA scheme for numerical treatment of many potential application arising in the fields of bioinformatics^42–44^, astro/plasma/atomic physics^45–47^, nonlinear circuit models^48–50^, fluid mechanics^51–56^, financial mathematics^57,58^ and COVID-19 virus models^59,60^.

## List of symbols

$$x,y$$: Cartesian coordinate
$$\overline{u},\overline{v}$$: Velocity components
$Cf_{x} ,Nu_{x}$: Skin friction, Nusselt number
$$C_{p}$$: Specific heat
$$Re_{x}$$: Reynold number
$T,T_{z} ,T_{\infty }$: Fluid, wall and ambient temperature
$$m$$: Shape factor
$V_{e}$: Stream stress velocity
$V_{w}$: Fluid stream velocity
$f,s,nf$: Fluid, solid, nanofluid
$\rho_{nf}$: Density of nanofluid
$\upmu _{nf}$: Dynamics viscosity of the nanofluid
*A*: Stretching ratio parameter
$$\mu_{nf}$$: Nanofluid viscosity
$$k$$: Thermal conductivity
$B_{0}$: Magnetic field component
$$\tau_{z}$$: Shear stress
$$\sigma$$: Electrical conductivity
$$\rho$$: Particle density
Pr: Prandtl number
M: Hartmann number
Gr: Grashof number
$$\uplambda $$: Viscosity parameter
$$\upsigma _{nf}$$: Electrical conductivity
$$k_{nf}$$: Thermal conductivity
